# Non-verbal social communication in individuals with eating disorders: an ethological analysis in experimental setting

**DOI:** 10.1007/s40519-022-01442-2

**Published:** 2022-07-13

**Authors:** Alessio Maria Monteleone, Giammarco Cascino, Valeria Ruzzi, Niccolò Marafioti, Luigi Marone, Roberta Croce Nanni, Alfonso Troisi

**Affiliations:** 1 Department of Psychiatry, University of Campania “Luigi Vanvitelli”, Largo Madonna Delle Grazie, 80138 Naples, Italy; 2 Department of Medicine, Surgery and Dentistry ‘Scuola Medica Salernitana’, Section of Neurosciences, University of Salerno, Salerno, Italy; 3 Department of Systems Medicine, University of Rome Tor Vergata, Rome, Italy

**Keywords:** Eating disorders, Non-verbal communications, Ethology, Trier social stress test, Experimental, Behavior

## Abstract

**Purpose:**

Evidence that social difficulties promote the development and the maintenance of eating disorders (EDs) derive from self-reported data and only partially from experimental tasks. This study objectively assessed non-verbal behaviors of individuals with EDs in a psycho-social stress scenario.

**Methods:**

Thirty-one women suffering from EDs (13 with anorexia nervosa and 18 with bulimia nervosa) and 15 healthy women underwent the Trier Social Stress Test (TSST), the paradigm of psycho-social stress, and were videotaped. Throughout the procedure, anxiety feelings were measured by the State-Trait Anxiety Inventory state subscale and saliva samples were collected to evaluate cortisol levels. Non-verbal behaviors were analyzed through the Ethological Coding System for Interviews and were compared between study samples through multivariate analysis of variance. Multivariate regression analyses were performed to assess the association between anxiety, cortisol and behavioral responses to TSST.

**Results:**

Women with EDs showed reduced submissiveness, flight (cutoff from social stimuli) and gesture compared to healthy peers during TSST. Submissiveness and flight behaviors were negatively associated with stress-induced anxiety, while TSST-induced anxiety and cortisol increases were positively associated with looking at the other’s face behavior in participants with EDs. In this population, cortisol reactivity was also positively associated with submissiveness and negatively with gesture.

**Conclusion:**

Women with EDs showed a hostile and freezing response to acute psycho-social stress: reduced submissiveness and flight may represent strategies to manage social anxiety. These findings confirm that the non-verbal behavior assessment provides complementary information to those derived from traditional measurements and suggests research and clinical implications.

**Level of evidence I:**

Evidence obtained from experimental study**.**

## Introduction

A crisis of the diagnosis in psychiatry has been recently advocated [1], based on the evidence that the current diagnostic systems are not enough adequate to draw treatment plan and to provide prognostic information [2–4]. In the eating disorder (ED) field, this is supported from the evidence that individuals with DSM-5 defined ED diagnoses are characterized by high occurrence of psychiatric comorbidity [5] and diagnostic cross-over [6, 7], which point to a problem of longitudinal diagnostic instability. Furthermore, there is a wide within-diagnosis variability in clinical presentation of EDs, especially in adolescence [8], and the Other Specified Feeding and Eating Disorders is the most common ED diagnosis, although it does not differ from anorexia nervosa (AN) or bulimia nervosa (BN) in terms of age of onset, comorbidity and treatment [9].

Novel classification systems describing psychopathology from a dimensional perspective have been proposed also for EDs. They include the Hierarchical Taxonomy of Psychopathology (HiToP) model [10–12] and the network theory [13]. The HiToP approach highlighted the role of internalizing symptoms in people with EDs [14], while the network theory showed high centrality of affective symptoms, self-esteem problems and interpersonal difficulties [15]. A complementary approach to these systems is represented by the RDoC project [16], which aims to identify the neurobiological bases contributing to the development of psychiatric symptoms. It describes five dimensional domains that should be evaluated through several levels of analysis. The “system for social process” is the RDoC domain which includes problems with social and emotional functioning. A few studies have utilized this design in EDs [17] and have shown self-reported social impairments [18–20] in terms of insecure attachment, impaired facial emotion recognition and communication, alexithymia, negative self-evaluation and social inferiority. More recently, experimental tasks have been employed and provided evidence of impaired emotional and biological responses to an acute social threat [21–23] as well as of compromised socio-cognitive processes (i.e., mentalization, imitation) [24]. However, most of the psychological measurements performed in these studies have been gained through indirect reports provided by the patients themselves while the evaluation of objective behavioral changes has been partially neglected in EDs.

Behavioral assessment may provide useful data for the definition of mental disorders [25]. Information derived from questionnaires and structured interviews often yielded different results from that provided by the objective and quantitative recording of patients’ behaviors [26–28]. Furthermore, behavioral ratings may allow researchers to do a more accurate evaluation of the association between behaviors and physiological measurements than that provided by subjective self-report and clinicians’ generated rating questionnaires [29]. This is even more interesting when considering the possibility to assess non-verbal behaviors during people interaction with social environment given that the inability to achieve biosocial goals (i.e., the need to affiliate or to attain an acceptable rank in the society) is considered at the core of many psychiatric conditions [30–32] including EDs [33].

This study aimed to employ the ethological approach to obtain an objective assessment of social functional impairment in individuals with EDs while exposed to an acute psycho-social challenge. Exploring social interactions through the investigation of facial expression, gesture and body language may provide new insights to conceptualize the psychopathology of EDs. Furthermore, we investigated the relationships of non-verbal behaviors with self-reported emotional response (i.e., anxiety) and physiological measures (i.e., cortisol) of stress response. Consistently with previous findings relative to social processing [18] and to the hyper-responsivity of fear circuits in EDs [34, 35] and considering the evidence from other psychiatric disorders [36], we hypothesized that individuals with EDs would show higher submissiveness and displacement than healthy peers. Given the exploratory nature of these analyses, we had no specific a priori hypotheses on the associations between the psychological, biological and behavioral components of the stress response in such a population.

## Methods

### Participants

This study was a secondary analysis of data including a sub-group of patients from a larger dataset of participants [21]. Patients were recruited from consecutive referrals to the ED outpatient centers of the University of Campania L. Vanvitelli and of the University of Salerno. Inclusion criteria were: (a) current diagnosis of AN, atypical AN or BN according to DSM-5 criteria; (b) age ≥ 18 years; (c) female gender; (d) absence of current or lifetime comorbid diagnoses of schizophrenia, bipolar disorder or substance abuse disorder; (e) no use of contraceptive pills; (f) absence of non ED-related severe physical disorders; (g) no use of psychoactive medications in the past 2 months; (h) no smoking cigarettes in the last 3 months. At admission to the ED unit, diagnostic assessment was performed by expert psychiatrists through a routine clinical interview and the employment of the Structured Clinical Interview for DSM-5 Disorders–Research Version to confirm the ED diagnosis and to investigate psychiatric comorbidity [37, 38].

Healthy women were also enrolled among health professionals and trainees at the recruiting centers. They underwent physical examination, routine medical interview, and the Mini International Neuropsychiatric Interview [39] to evaluate if they met the following inclusion criteria: lack of physical and mental disorders, regularly menstruating, and drug-free.

The study was approved by the ethics committee of the University of Salerno Campania Sud (number: 18_r.p.o.14/02/2017) and was carried out in accordance with the Declaration of Helsinki for experiments involving humans. All participants gave their written consent after being fully informed of the nature and procedures of the study.

### Protocol

The study procedure was conducted at referral to the ED services (before entering specific treatment programs).

Participants underwent two research sessions scheduled in two consecutive days.

During the first test day, they filled in the following self-report questionnaires: the Eating Disorder Inventory-2 (EDI-2) [40], assessing ED-related psychopathology, and the State-Trait Anxiety Inventory (STAI) [41], evaluating state and trait anxiety.

During the second test day, participants were exposed to the TSST procedure. The test was carried out in the afternoon to adequately explore the impact of stress on the hypothalamus–pituitary–adrenal (HPA) axis response, in accordance with Kudielka et al. suggestions (2009). Considering that sex steroids may impact the endogenous cortisol response [42], menstruating women underwent the procedure in their follicular phase (i.e., within 7 days from the starting of menses). In this way the plasma estrogen milieu in menstruating women was as close as possible to that of non-menstruating women suffering from AN.

To undergo the TSST procedure, the participants were asked to refrain from food, drink (except water), and physical exercise after breakfast. At the beginning of the session, they were received in a room (number 1) for 20 min to perform a resting period. During the first 10 min, the operator described them the test procedure which consisted of giving a 5-min mock job interview to an unknown panel on personal eligibility for a job, outlining personal skills, followed by a 5-min mental arithmetic task performed out loud. This task consisted of serially subtracting 13 from 2034, as quickly as possible, still in front of the interviewers. In case of a mistake, the participant was asked to start over from the beginning. The interviewers were one man and one woman sitting behind a desk in another room (number 2) and maintaining a neutral evaluative facial expression. Participants were told that their performance would have been videotaped for analysis of the non-verbal behavior. At the end of the procedure explanation, participants had 10 min to plan the speech. Afterwards, they were conducted in the room number 2 where they started the performance. At the end of the tasks, they went back to room number 1 where they had a 50 min rest and could relax through magazine readings.

### Psychological and biological response assessment during the TSST

The STAI-state subscale [41] was used to investigate anxious feelings throughout the experimental procedure. The participants were asked to complete this scale at the end of the resting period (*T* = − 20), immediately before starting the test (*T* = 0), at the end of the test (*T* = 10) and 50 min (*T* = 60) after the end of the test. For the purpose of this study (i.e., assessing the relationship between anxiety and non-verbal behavior performed during the tasks), the anxiety area under the curve relative to the ground (AUCg) and the AUC with respect to the increase (AUCi) were calculated including T = − 20, T = 0 and T = 10 time-points [43]. Internal consistency across the items used in the present study ranged between 0.84 and 0.87 at the three time-points.

Saliva samples were collected in Salivette tubes (Sarstedt; Rommelsdorft, Germany) at the end of the resting period (*T* = − 20), immediately before starting the TSST (*T* = 0), at the end of the TSST (*T* = 10) and 10 (*T* = 20), 30 (*T* = 40) and 50 min (*T* = 60) after the end of the test. Saliva was centrifugated and stored at  − 20 °C until being tested for cortisol concentration that was determined through an enzyme immunoassay method, using a commercially available ELISA kit (Biochem Immunosystem, Milan, Italy). Intra- and inter-assay coefficients of variation were less than 8% and 8.7%, respectively. With the aim to assess the association between cortisol response and non-verbal behavior performed during the tasks, the cortisol AUCg and AUCi were calculated including *T* = − 20, *T* = 0, *T* = 10 and *T* = 20 time-points [43].

### Non-verbal behavior assessment during the TSST

Non-verbal behavior data were available for 31 participants with EDs (8 with AN restricting subtype, 5 with AN purging subtype and 18 with bulimia nervosa) and 15 healthy women.

The non-verbal behavior of study participants was evaluated using the Ethological Coding System for Interviews (ECSI; Troisi, 1999). The ECSI comprises 37 different patterns of behavior, particularly facial expressions, and head movements as well as body posture, gestures, and whole-body movements. This coding system was specifically designed for measuring non-verbal behavior during interviews.

The interviews were videotaped with a camera located behind the interviewers and adjusted so that the subject’s face and trunk were in full view. One trained observer examined the videotapes and scored subjects’ behavior according to the behavioral taxonomy of the ECSI. During examination of the videotapes, the volume of the player was turned mute. The overall duration of the videotaped part of the interview was 10 min and corresponded to the test procedure (namely the 5-min mock job interview and the 5-min mental arithmetic task).

Before the beginning of the study, the observer was trained until she reached an adequate level of inter-observer reliability (i.e., a κ coefficient of at least 0.70 for each behavior pattern) with decoding by a qualified observer as the standard reference. Assessment of inter-observer reliability was based on a sample of 30 interviews not including those with the subjects of this study. Recording method was one-zero sampling. The recording session was divided into successive 15-s sample intervals. The instant of time at the end of each sample interval, referred to as the “sample point”, was identified by a beeper. On the instant of each sample point, the observer recorded whether the behavior pattern had occurred during the preceding sample interval. The score of each behavior for each subject was expressed as the proportion of all sample intervals during which that behavior occurred. One-zero scores are highly correlated with both frequency and duration measures of the same behavior, which means that they give a composite measure of “amount” of behavior. Using a short sample interval (i.e., 15 s), the scores obtained using one-zero sampling and continuous recording are highly correlated.

The interpretation of the social and emotional meaning of the individual ethological profile is based on the scores on the following behavioral categories. Eye contact (alias look at) represents an important aspect in social interactions. The amount of eye contact usually expresses attention and involvement. Affiliation includes patterns of behavior that invite and positively reassure social interaction. Submissive behaviors are appeasement signals and are used to prevent aggressive responses in the interlocutor. Flight embraces behavioral patterns that are used to cut off social stimuli that are perceived adverse. Assertion describes a set of behaviors indicating low levels of aggression and disagreement. Gesture is used to illustrate or emphasize the meaning of spoken language. Displacement activities comprise behaviors of self-manipulation (e.g., scratching) or manipulation of objects (fumbling). These patterns of behavior are strongly suggestive of motivational conflict. Relaxation includes behaviors indicating low levels of emotional arousal.

### Statistical analyses

Descriptive statistics and multivariate analysis of variance (MANOVA) were conducted in SPSS v. 27. A power analysis was conducted using GPower with alpha set at 0.05, power set at 80% and aiming for a low-medium effect size (*d* = 0.4). This suggested a minimum sample size of 46 participants.

A MANOVA was carried out to assess differences in non-verbal behavior (ECSI scores) between participants with EDs and healthy women along a linear combination of ECSI variables by taking in consideration the covariation among the different dependent variables. To consider the effect of malnutrition on social functioning [18, 20], the body mass index (BMI) was included as covariate in the model.

To investigate the associations between anxiety/cortisol responses to TSST and non-verbal behavior during the TSST, multivariate regression analyses were run in each group of participants. The BMI, the AUCg and the AUCi of salivary cortisol and of anxiety were included as predictors, while the non-verbal behaviors scores were included as dependent variables. Multivariate regression analyses were conducted using *lavaan* package [44] in R, Version 3.6.1 [45].

## Results

Demographic and clinical characteristics of the sample are reported in Table 1.

**Table 1:** Demographic and clinical characteristics of study sample (mean ± SD)

	Women with eating disorder (*n* = 31)	Healthy women (*n* = 14)
Age	26.7 ± 8.7	24.2 ± 2.8
Body mass index	23.2 ± 7.6	21.5 ± 2.8
Age at onset	18.5 ± 5.4	NA
Illness duration (years)	8.7 ± 8.1	NA
Education (years)	14.7 ± 2.9	13.9 ± 1.8
Marital status (single, %)	28 (90)	13 (87)

The results of MANOVA are reported in Table 2.

**Table 2:** Differences in non-verbal behaviors between women with eating disorders (ED) and healthy women through multivariate analysis of variance (MANOVA)

	Women with ED (*n* = 31) Mean ± SD	Healthy women (*n* = 15) Mean ± SD	*F*_1, 44_	*p*
Look at	15.74 ± 5.63	15.33 ± 5.34	0.05	0.82
Affiliation	10.81 ± 6.91	12.33 ± 6.37	0.52	0.47
Submissiveness	7.55 ± 4.22	12.07 ± 6.72	7.79	0.008
Flight	18.35 ± 8.67	24.4 ± 10.03	7.63	0.008
Assertion	32.0 ± 5.92	36.93 ± 5.13	0.69	0.41
Gesture	10.52 ± 6.02	9 ± 5.15	4.73	0.03
Displacement	4.26 ± 5.21	8.2 ± 6.79	0.26	0.61
Relaxation	12.23 ± 9.46	13.6 ± 6	0.01	0.98
Body mass index	23.2 ± 7.6	21.5 ± 2.8	0.19	0.66

**Fig. 1:**
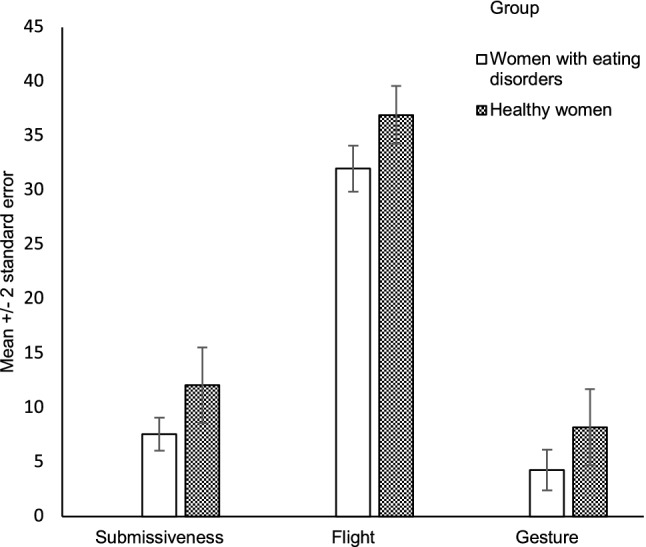
Non-verbal behaviors during the Trier Social Stress Test compared between women with eating disorders and healthy women

MANOVA for non-verbal behaviors during the exposure to TSST disclosed a significant effect of diagnostic group (*V* = 0.36, *F*_9,36_ = 2.5, *p* = 0.02) but not of BMI (*V* = 0.08, *F*_9,36_ = 0.19, *p* = 0.6). This means that, although ECSI scores co-varied significantly, they significantly differed between women with EDs and healthy peers. In comparison to healthy women, participants with EDs showed reduced occurrence of submissiveness (*p* = 0.008) and flight (*p* = 0.008) behaviors and less gesture (*p* = 0.03). Figure 1 shows the submissiveness, flight and gesture scores of the two study groups.

In participants with EDs, the multivariate regression model showed that submissiveness was positively associated with cortisol AUCg (β = 0.35, *p* = 0.03), cortisol AUCi (β = 0.35, *p* = 0.029) and BMI (β = 0.48, *p* < 0.01), but negatively with anxiety AUCg (β = − 0.59, *p* < 0.01). In addition, look at the other’s face was positively associated with cortisol AUCi (β = 0.42, *p* = 0.02) and anxiety AUCi (β = 0.49, *p* = 0.01); flight was negatively associated with anxiety AUCg (β = − 0.52, *p* = 0.02) while gesture with cortisol AUCi (β = − 0.48, *p* = 0.04).

In healthy women, the multivariate regression model showed that cortisol AUCg was positively associated with submissiveness (β = 0.72, *p* < 0.01) and negatively associated with gesture (β = − 0.61, *p* < 0.01) and with relaxation (β = − 0.47, *p* = 0.04). Anxiety AUCi was negatively associated with relaxation (β = − 0.48, *p* = 0.04).

## Discussion

This study aimed to explore non-verbal behaviors during the exposure to a psycho-social stress in individuals with EDs through an ethological approach. Compared to healthy peers, women with EDs showed lower occurrence of submissive and flight behaviors and reduced gesture. In women with EDs, the anxiety experienced during the stress exposure was negatively associated with submissive and flight behaviors, while TSST-induced anxiety and cortisol increases were positively associated with looking at the other’s face behavior. In this population, stress-induced cortisol reactivity was also positively associated with submissiveness and negatively associated with gesture.

To the best of our knowledge, this is the first study employing an ethological approach to investigate non-verbal behaviors during social interactions in individuals with EDs. Our first hypothesis was not confirmed, since no significant difference was detected in displacement behaviors between women with EDs and healthy peers whereas in other psychiatric disorders those behaviors were more frequent [36] and were associated with anxiety experienced during social interactions [29]. In addition, in contrast with our hypothesis, submissive as well as flight behaviors showed lower frequency in participants with EDs and were negatively associated with anxiety feelings during the social stress exposure. Literature studies based on self-report measures showed heightened submissive behaviors and feelings of social inferiority [18] that were positively associated to the severity of ED-related symptoms in individuals with EDs [46, 47]. Experimental studies also found heightened vigilance to social rank cues with higher negative expectations about others’ beliefs [48]. The discrepancy between literature data and current findings may reflect the inconsistency between objective assessment and self-reported recording of patients’ behavior as already reported in other psychiatric disorders [26–28]. Submission is a prosocial behavior that allows people to prevent hostile responses. The present findings may point to a reduced tendency of individuals with EDs to engage in social relationships while experiencing high levels of anxiety. The neutral faces of interviewers in the TSST represent an ambiguous scenario that is usually negatively interpreted by individuals with EDs [49]: this kind of social scenario may promote a hostile response.

Given the heightened facial avoidance [18] and the increased anxiety [50] experienced in social contexts by individuals with EDs, a higher occurrence of flight behaviors would have been expected in this population compared to healthy peers. Indeed, flight is a temporarily disengagement from the interaction that is needed to disconnect from social stimuli perceived as aversive [51]. A possible explanation of our findings is that individuals with EDs tend to assume a more freezing posture to monitor the other’s behavior. Indeed, we also found that gazing at the interviewer’s face was positively associated with anxiety and cortisol increases during the stress exposure. This hypothesis is in line with the increased attentional bias to threatening stimuli (i.e., faces expressing rejection) previously observed in this population [50, 52]: indeed, detection and attention to potential threats are the first steps in the development of threat-coping strategies and tend to occur rapidly, automatically and outside of conscious control. Furthermore, the current findings may point to an attempt to meet other people’s expectations about the self, which has been described as a safety strategy towards social anxiety [53].

Interestingly, the assessment of non-verbal behaviors also displayed reduced gesture in participants with EDs compared to healthy peers. An experimental study [54] showed that individuals with EDs tend to respond coldly to the other’s feedback. A recent meta-analysis [55] and an experimental study [56] showed that individuals with EDs have reduced emotional facial expression while looking either pictures or film clips evoking positive and negative emotions. Caglar-Nazali et al.’s meta-analysis (2014) also found poorer facial communication in participants with EDs. The gesture assessed in this study also includes hand movements and points to the subject’s communicative effort in social interactions [29]. In the light of literature data, it is possible to hypothesize that our findings show either a decreased social engagement of individuals with EDs or an increased fear of expression and social inhibition when feeling to be criticized [19, 57]. The lack of clarity of proper emotions that characterizes this population [58] may also contribute to the lower gesture.

Limitations of this study need to be acknowledged. First, the low size of the sample affects the reliability of our findings and does not allow to perform the analyses in each diagnostic sub-group. Future studies are needed to confirm the current findings and to explore potential differences between homogeneous ED subgroups (i.e., the AN restricting type) and healthy peers also considering variables affecting social stress response (namely, autistic traits or early adverse experiences [59]). Second, the use of a laboratory procedure, the TSST, although well validated and highly used [60], yielded data relative to non-verbal behaviors that need to be considered an indirect measurement of those occurring in the real-life. Third, the assessment of other components of the endogenous stress response system (i.e., the sympathetic nervous one) could be helpful to evaluate the association between non-verbal behaviors and physiological stress response.

However, this study has some strengths such as the use of an experimental task that has been highly recommended to improve the understanding of ED psychopathology [61] and the employment of the ethological approach to assess non-verbal behaviors.

### Clinical implications and conclusions

This is the first study in EDs that investigates the three components of the stress response [62]: the subjective experience, the physiological reaction, and the behavioral response. We showed that the reduction of gesture, submissive and flight behaviors may represent the behavioral attempt to downregulate the heightened [21] anxiety experienced by participants with EDs in comparison to healthy peers. Furthermore, an association between the biological (cortisol) response and objectively assessed behaviors (submissiveness, gesture and gazing another’s face) was observed both in women with EDs and in healthy women, while no significant association between physiological and psychological measurements was reported in previous studies in ED people [63] and in mixed psychiatric populations (for a review see Campbell & Ehlert, 2012). This supports the idea that objectively assessed non-verbal behaviors may be coupled with physiological measures more strongly than subjective psychological experiences.

The differences observed in non-verbal behaviors of individuals with EDs may have a profound impact on their social functioning by disrupting their communications and increasing perceived stress levels [65]. Indeed, non-verbal behaviors such as submissiveness and flight are needed to induce the other to deescalate and refrain from perpetrating harm: the reduction of these behaviors while experiencing social distress may be dysfunctional. These alterations may affect social interactions contributing to isolation [66] and reduced support perceived by these subjects [67]. This is important for the triggering of the illness given that ED behaviors and weight loss have been described as an attempt to avoid the competition for social status (a “losing strategy”) [68] and to distract from the pain of social isolation [33, 69]. In addition, illness maintenance is promoted by social difficulties including impaired interactions with carers [70].

Our findings support the importance of the ethological approach and of the objective assessment of social behaviors as means to improve the knowledge of psychopathology through more reliable and complementary information that can be added to those derived from self-report and clinical generated rating scales. Furthermore, the observed discrepancy between the literature suggested subordinate psychological status and the objectively assessed hostile social behavior of individuals with EDs may propose novel treatment strategies aiming to improve the patients’ awareness of their non-verbal communications in social stress scenarios.

### What is already known on this subject?

Social difficulties promote either the development or the maintenance of eating disorder psychopathology. However, this evidence largely derives from self-reported data and the objective assessment of social behaviors has been neglected.

### What does this study add?

People with eating disorders show reduced submissiveness, flight and gesture in response to acute social stress. Although these behaviors represent strategies to manage social anxiety, they affect social communication and may contribute to social difficulties of these individuals. Objectively assessed non-verbal behaviors are associated with physiological stress measures and provide novel information in comparison to those derived from self-report measurements.

## Data Availability

The data that support the findings of this study are available from the corresponding author upon reasonable request.
